# The potential for flower nectar to allow mosquito to mosquito transmission of *Francisella tularensis*

**DOI:** 10.1371/journal.pone.0175157

**Published:** 2017-05-09

**Authors:** Adam Kenney, Austin Cusick, Jessica Payne, Anna Gaughenbaugh, Andrea Renshaw, Jenna Wright, Roger Seeber, Rebecca Barnes, Aleksandr Florjanczyk, Joseph Horzempa

**Affiliations:** Department of Natural Sciences and Mathematics, West Liberty University, West Liberty, West Virginia, United States of America; Metabiota, UNITED STATES

## Abstract

*Francisella tularensis* is disseminated in nature by biting arthropods such as mosquitoes. The relationship between mosquitoes and *F*. *tularensis* in nature is highly ambiguous, due in part to the fact that mosquitoes have caused significant tularemia outbreaks despite being classified as a mechanical vector of *F*. *tularensis*. One possible explanation for mosquitoes being a prominent, yet mechanical vector is that these insects feed on flower nectar between blood meals, allowing for transmission of *F*. *tularensis* between mosquitoes. Here, we aimed to assess whether *F*. *tularensis* could survive in flower nectar. Moreover, we examined if mosquitoes could interact with or ingest and transmit *F*. *tularensis* from one source of nectar to another. *F*. *tularensis* exhibited robust survivability in flower nectar with concentrations of viable bacteria remaining consistent with the rich growth medium. Furthermore, *F*. *tularensis* was able to survive (albeit to a lesser extent) in 30% sucrose (a nectar surrogate) over a period of time consistent with that of a typical flower bloom. Although we observed diminished bacterial survival in the nectar surrogate, mosquitoes that fed on this material became colonized with *F*. *tularensis*. Finally, colonized mosquitoes were capable of transferring *F*. *tularensis* to a sterile nectar surrogate. These data suggest that flower nectar may be capable of serving as a temporary source of *F*. *tularensis* that could contribute to the amplification of outbreaks. Mosquitoes that feed on an infected mammalian host and subsequently feed on flower nectar could deposit some *F*. *tularensis* bacteria into the nectar in the process. Mosquitoes subsequently feeding on this nectar source could potentially become colonized by *F*. *tularensis*. Thus, the possibility exists that flower nectar may allow for vector-vector transmission of *F*. *tularensis*.

## Introduction

The bacterium *Francisella tularensis* is the causative agent of the potentially lethal disease tularemia and is commonly spread in nature by biting arthropods [1–4]. Among the known vectors of tularemia, ticks have been the most extensively studied [5–7]. In addition to ticks, mosquitoes can be arthropod vectors of *F*. *tularensis*, especially in Scandinavian countries [8–11]. Because mosquitoes can reproduce rapidly, this escalates the possibility of emergent outbreaks of tularemia. Despite these phenomena, mosquitoes have not been thoroughly evaluated as vectors of *F*. *tularensis* and, as such, there may be yet unexplored factors contributing to the ability of these insects to maintain and spread tularemia in nature.

Mosquitoes are considered to be the primary transmission route of tularemia in Scandinavian countries based on epidemiological data and clinical reports [8, 12–15]. There have been several notable tularemia outbreaks linked to an increase in mosquito prevalence, indicating that these arthropods are important vectors of *F*. *tularensis* [12, 16]. Specifically, *Aedes cinereus* mosquitoes have been reported as vectors for tularemia as early as 1941 [17]. Since this time, more than 10 different mosquito species have been identified to harbor and potentially transmit *F*. *tularensis* [18]. Mosquitoes have been tentatively classified as mechanical vectors of tularemia and are thought to only transmit this disease transiently [2, 19]. However, *F*. *tularensis* can persist through stages of mosquito development suggesting that mosquitoes are capable of perpetuating *F*. *tularensis* in nature more efficiently than a typical mechanical vector [18, 20, 21]. Nevertheless, the possibility exists that ecological parameters of the mosquito lifestyle may contribute to large mosquito-borne outbreaks of tularemia.

When acquiring a blood meal from an infected mammal, female mosquitoes could potentially acquire the infectious microbe. When acting as a mechanical vector, feeding mosquitoes transmit infectious microbes during subsequent blood meals. Between blood meals, female mosquitoes occasionally feed on flower nectar (male mosquitoes do not feed on blood, only nectar and other plant juices) [22]. This intermittent feeding allows for the possibility that a colonized mosquito could deposit infectious microbes into flower nectar. Consequently, any mosquito feeding on this newly inoculated nectar could ingest pathogenic microbes and become a carrier with the potential to infect mammalian hosts. In this study, we test if nectar could serve as a potential temporary source of *F*. *tularensis*. The plausibility of this hypothesis depends on the ability of *F*. *tularensis* to survive in flower nectar as well as the capability of mosquitoes to ingest and transfer the bacterium from the nectar of one flower to another.

Here, we show that flower nectar supports survival of *F*. *tularensis* similarly to a nutrient-rich growth medium. Colonies of *F*. *tularensis* were recovered from mosquitoes that fed on inoculated nectar, suggesting that *F*. *tularensis* bacteria residing in nectar are capable of colonizing mosquitoes. Further, colonized mosquitoes were capable of inoculating sterile nectar with *F*. *tularensis*. We propose that flower nectar could act as a temporary source of *F*. *tularensis* and that this may increase the ability of mosquitoes to spread this bacterium in nature.

## Materials and methods

### Bacterial cultivation

To cultivate *Francisella tularensis* Live Vaccine Strain (LVS) (a gift from Karen Elkins), tryptic soy broth with 0.1% cysteine (TSBc) was inoculated with a loop full of bacteria from a Chocolate II Agar plate (GC II agar with 1% hemoglobin and supplemented with IsoVitaleX^tm^ [BD]). The broth culture was incubated overnight at 37°C with agitation to an OD_600_ ≥ 3.

### Rearing/Maintenance of mosquitoes

A single preparation of *Aedes aegypti* eggs (BEI resources; Centers for Disease Control and Prevention) was placed in 500 ml water in the bottom section of a Mosquito Breeder chamber (BioQuip). A liver powder slurry was the food source provided for larvae [23]. After reaching adulthood (approx. 2–3 weeks in untreated distilled water; 1–2 weeks in water that was boiled, cooled, then saturated with mosquito feed), mosquitoes were fed 30% sucrose solution for sustenance until beginning of experimentation. Mosquitoes were provided horse blood (defibrinated, Hemostat Laboratories) to promote female fertility when necessary.

One ml of horse blood was added to a 1.5 ml microcentrifuge tube. The opening of the tube was sealed tightly with paraffin to simulate a mammalian membrane. Blood was incubated and presented in proximity to boiled water to provide heat and water vapor, facilitating feeding. Newly deposited eggs were recovered on Whatman filter paper and stored at 4°C until needed.

### Extraction of nectar

Mature flowers from yellow squash plants, *Cucurbita pepo*, were opened and glass capillary tubes were inserted into the base of the pistil of the flower. The entire nectar volume per flower was extracted and collected in a centrifuge tube. Nectar was stored at -20°C.

### Determination of survival in nectar

*F*. *tularensis* LVS cultivated to stationary phase in TSBc was added to yellow squash nectar, 30% sucrose, TSBc, or distilled water at a concentration of 10^7^–10^8^ CFU/ml. These suspensions remained stationary and were incubated at 22°C. Daily, for seven days, bacterial suspensions were serially diluted and plated on Chocolate II agar to determine CFU. After 72 hours of incubation at 37°C, 5% CO_2_, colonies were counted and CFU/ml were calculated.

### Determination of mosquito colonization from bacteria residing in nectar surrogate

Microcentrifuge tubes containing 30% sterile sucrose solution or 30% sucrose solution inoculated with *F*. *tularensis* (10^7^–10^8^ CFU/ml as indicated previously) were placed in separate mosquito chambers. The tubes were left open and placed on their sides to allow mosquitoes to access the liquid. Mosquitoes were exposed to and able to feed on solutions for 24 hour time increments for 6 days. After each time period, the suspensions were replaced with freshly prepared solutions.

As many as six mosquitoes per group were extracted indiscriminately each day. Mosquito rearing chambers were temporarily placed in a cold room to slow the activity of these insects. Once mosquitoes stopped moving, insects were aseptically removed, washed with gentamicin to kill surface bacteria (100 μg/ml), placed individually into sterile phosphate-buffered saline (PBS) solution, and homogenized with an Omni tissue homogenizer. One ml of mosquito homogenate (the entire mosquito) was spread plated, in aliquots of 200 μl, on media selective for *F*. *tularensis* (Chocolate II agar containing antibiotics Vancomycin [12.5 μg/ml], Ampicillin [100 μg/ml], and Polymixin-B [100 μg/ml]) and incubated for 72 hours at 37°C, 5% CO_2_. DNA was extracted from bacteria producing colonies with a similar morphology to *F*. *tularensis*. The extracted DNA was subjected to PCR using primers specific for the *macrophage growth locus A* (*mglA*) of *Francisella* bacteria using the following primer pair: mglAF, 5’-ACTGGAATTCGATATAGTCCGCATGATCCTTC; mglAR, 5’-GTCAGCTAGCGGTACTATAACACCT TCATACTCG [24]. Agarose gel electrophoresis was used to compare amplicon bands produced from bacteria isolated from mosquitoes with bona fide *F*. *tularensis* DNA. To confirm that PCR specifically identified *F*. *tularensis*, cell extracts of isolated bacteria were subjected to Western blotting using an anti-IglC monoclonal antibody (BEI resources) as a probe. Western blotting was conducted in a similar fashion as previously described [25, 26].

### Retrieval of *F*. *tularensis* from previously sterile nectar surrogate after deposition of bacteria by mosquitos

Tubes holding a 30% sucrose solution inoculated with *F*. *tularensis* (107–10^8^ CFU/ml) were placed within insect chambers containing twenty-five adult, *A*. *aegypti* mosquitoes. These tubes remained within the chamber for the entirety of the experiment. Additional containers (2 cm dishes) holding sterile 30% sucrose were included in these insect chambers as well. This sterile sucrose was replaced and assayed daily for the presence of *F*. *tularensis*. After retrieval, the sterile sucrose solution was plated on *Francisella*-selective media to assess if any bacteria had been transferred from the inoculated mosquitoes. DNA was extracted from colonies that had been recovered exhibiting a similar morphology to *F*. *tularensis*, and this genomic material was subjected to PCR in a similar fashion to the previously described procedure.

Control experiments were conducted in a similar fashion except these chambers were never introduced to *F*. *tularensis*. *F*. *tularensis* LVS was never detected in the sterile sucrose solution in the absence of mosquitoes or in the control chambers. This experiment was repeated twice (three total replicates).

## Results and discussion

### *F*. *tularensis* survival in nectar and nectar surrogates

**Fig 1:**
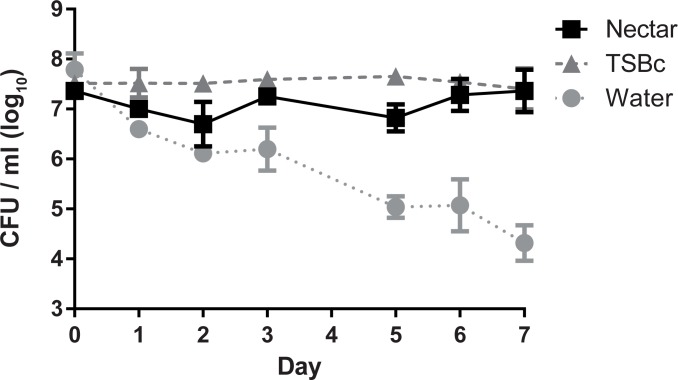
*F*. *tularensis* survives in flower nectar. *F*. *tularensis* bacteria were incubated in TSBc, yellow squash nectar, or water. Bacterial suspensions were incubated at 22°C, serial diluted and plated to determine CFU at the indicated time points. Graphed values represent mean CFU ± SE of three combined independent experiments. In some cases, error bars are smaller than the graph symbols. Differences in the average number of *F*. *tularensis* LVS recovered (CFU/mL) over the course of the assay were determined by repeated measures two-way ANOVA with Tukey’s multiple comparison test as a post hoc analysis comparing the change in bacterial burden between experimental, positive control, and negative control groups (ANOVA, P < 0.0001; post hoc, Nectar vs. TSBc not significantly different; Nectar vs Water, P < 0.001; TSBc vs. Water, P < 0.001).

Between blood meals, mosquitoes feed on flower nectar [22]. The possibility exists that mosquitoes carrying *F*. *tularensis* could inoculate flower nectar during these intermittent feedings. To test this possibility, we first sought to determine whether *F*. *tularensis* could survive in flower nectar. To test this, yellow squash flower nectar was inoculated with *F*. *tularensis* LVS and viability of the bacteria was determined for 7 days, the typical nectar-bearing lifespan of a flower. Nectar is typically produced in low abundance and can be difficult to harvest adequate quantities; therefore yellow squash was used here because the flowers of these plants generate recoverable amounts of nectar (personal communication with Sam Droege, USGS Native Bee Inventory and Monitoring Lab). Data are represented as CFU/μl as this volume represents that of a typical nectar meal of an *A*. *aegypti* mosquito [27]. After 7 days of incubation, there were similar concentrations of *F*. *tularensis* LVS in nectar and TSBc, a nutrient-rich growth medium known to support *F*. *tularensis* LVS viability and growth; however, there was a reduced concentration of *F*. *tularensis* LVS in water (Fig 1). The modest level of growth in the TSBc can be attributed to the incubation temperature (22°C) [28]. These data indicate that *F*. *tularensis* LVS is capable of surviving in nectar during the longevity of a typical flower. Moreover, these results are consistent with the hypothesis that flower nectar is a plausible temporary source of *F*. *tularensis* allowing for mosquito to mosquito transmission. This experiment was designed to determine whether bacteria are capable of surviving in nectar over the course of the lifespan of a hypothetical flower (1 week). However, the concentration of *F*. *tularensis* bacteria used here is presumably much higher than what could be achieved in nature through natural inoculation. Consequently, we wanted to determine whether the high concentration of bacteria contributed to survival. In two separate experiments, squash nectar was inoculated with *F*. *tularensis* LVS bacteria at a low concentration of 6.45 (±1.76) CFU/μl. After 7 days of incubation, viable bacteria were recovered at a concentration of 0.2 CFU/ μl. This suggests that the higher bacterial concentration in Fig 1 may have contributed to the survival of *F*. *tularensis* LVS in the nectar. However, even at a low concentration, *F*. *tularensis* bacteria were able to survive for one week in flower nectar. This result suggests that flower nectar is capable of supporting *F*. *tularensis* survival.

**Fig 2:**
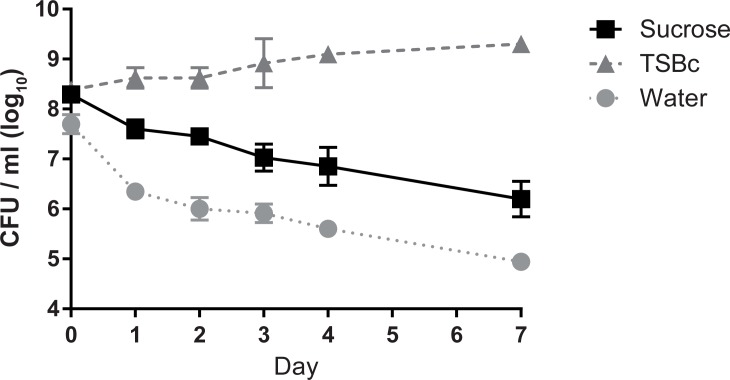
*F*. *tularensis* exhibits limited survival in a sucrose solution, a nectar surrogate. *F*. *tularensis* bacteria were incubated in TSBc, 30% sucrose, or water. Bacterial suspensions were incubated at ambient temperature, serial diluted and plated to determine CFU at the indicated time points. Graphed values represent mean CFU ± SE of four combined independent experiments. In some cases, error bars are smaller than the graph symbols. Differences in the average number of *F*. *tularensis* LVS recovered (CFU/mL) over the course of the assay were determined by repeated measures two-way ANOVA with Tukey’s multiple comparison test as a post hoc analysis comparing the change in bacterial burden between experimental, positive control, and negative control groups (ANOVA, P < 0.0001; post hoc, 30% Sucrose vs. TSBc, P < 0.001; 30% Sucrose vs. Water; P < 0.001; TSBc vs. Water, P<0.001).

Because collecting enough nectar for multiple laboratory trials was challenging, we aimed to formulate a nectar surrogate that could be routinely produced in high quantities to use in future experiments. Although sugar concentrations vary in nectar, we decided to use a 30% sucrose solution because that concentration is both easily suspended in solution and falls within the normal range of concentration of sucrose in nectar [23, 29]. *F*. *tularensis* LVS bacteria exhibited significantly greater viability in the 30% sucrose solution compared to the water control (Fig 2). However, the survival in 30% sucrose decreased over time and was significantly lower than that of *F*. *tularensis* LVS in TSBc (Fig 2). These data suggest that flower nectar contains additional components that enhance survival of *F*. *tularensis* LVS over time. Nonetheless, 30% sucrose solution was retained as a nectar surrogate because *F*. *tularensis* LVS exhibited adequate viability in this solution over the longevity of a typical flower (7 days).

### Colonization of mosquitoes by *F*. *tularensis* from a nectar surrogate

**Fig 3:**
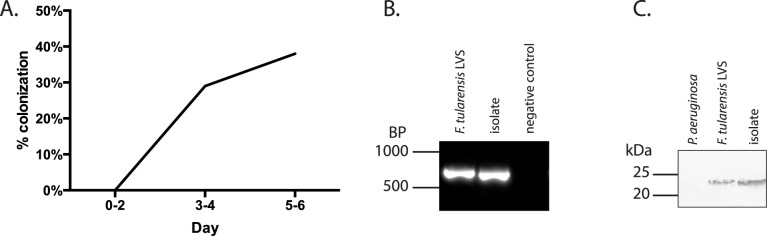
Mosquitoes become colonized with *F*. *tularensis* after interacting with flower nectar surrogate. **A.** Tubes holding a 30% sucrose solution inoculated with *F*. *tularensis* were placed within insect chambers for 6 days. As many as six mosquitoes per group were extracted daily, washed with gentamicin to kill surface bacteria, homogenized and plated on media selective for *Francisella*. Data shown represent the percentage of mosquitoes colonized with *F*. *tularensis* LVS (recovery of at least one CFU) on the days indicated and are a combination of three independent experiments. **B.** DNA was extracted from bacteria isolated from mosquito homogenates plated on media selective for *F*. *tularensis*. Only colonies that produced a similar morphology to *F*. *tularensis* were selected. The extracted DNA was subjected to PCR using primers specific for *mglA* of *Francisella* sp. Agarose gel electrophoresis was used to compare amplicons produced from bacteria isolated from mosquitoes to those generated from bona fide *F*. *tularensis* DNA. PCR from only one isolate is shown for simplicity, but all other colonies with similar morphologies produced a similar amplicon band (not shown). PCR reactions lacking *Francisella* template DNA did not produce amplicons (negative control). **C.** Cell extracts from isolates were separated by SDS-PAGE and electroblotted onto nitrocellulose. Following blocking, Western blots were probed with an anti-IglC monocolonal antibody. An alkaline-phosphatase anti mouse secondary antibody was used for detection.

We next aimed to determine if feeding mosquitoes could become colonized from bacteria inhabiting the nectar surrogate (30% sucrose). The sucrose solution was used here because we were unable to acquire adequate quantities of flower nectar for multiple replicate experiments. In addition, authentic, unprocessed flower nectar is not readily available from commercial sources. *A*. *aegypti* mosquitoes were utilized here as a laboratory model as others have done previously [18]. These mosquitoes are the closest relatives to *A*. *cinereus* (a mosquito known to transmit tularemia) that were available either commercially or from governmental repositories. Mosquitoes were exposed to nectar surrogate with or without *F*. *tularensis* LVS and allowed to feed on this material. At 24 hour intervals, mosquitoes were individually homogenized, and this material was plated on a medium selective for *F*. *tularensis*. As early as three days after the first exposure, colonies that resembled *F*. *tularensis* color and morphology were recovered from homogenates of mosquitoes that interacted with inoculated nectar surrogate (Fig 3A). Mosquitoes producing at least 1 CFU of *F*. *tularensis* LVS were considered to be colonized in these experiments. Although the total bacterial burden varied among the mosquitoes, all colonized mosquitoes contained fewer than 10^2^ *F*. *tularensis* LVS CFU. To verify that the recovered bacteria were *F*. *tularensis* LVS genomic DNA was subjected to PCR using primers specific for *F*. *tularensis mglA*, and the amplicon that had been produced was compared to one generated using bona fide *F*. *tularensis* DNA via gel electrophoresis. PCR using DNA from the isolated colonies produced a band with a similar size to that of the authentic *F*. *tularensis*, whereas the reaction lacking a template did not produce an amplicon (Fig 3B). Moreover, in a separate experiment, cell lysates of bacteria isolated from mosquitoes were subjected to western blotting in which an antibody specific for *F*. *tularensis* IglC was used as a probe (BEI resources) (Fig 3C). This blot revealed that isolated bacteria produced a protein band that reacted with this antibody at a similar apparent molecular weight to that produced by *F*. *tularensis* LVS, whereas *Pseudomonas aeruginosa* produced no protein band. Together, these results indicated that the bacteria recovered from the mosquitoes were *F*. *tularensis* LVS. *F*. *tularensis* LVS bacteria were not recovered from the control group mosquitoes fed un-inoculated nectar surrogate solution. These data suggest that if mosquitoes ingest/interact with *F*. *tularensis* from flower nectar in nature, these insects could be subsequently colonized.

### Transfer of *F*. *tularensis* bacteria by mosquitoes from one nectar surrogate to another

Finally, we aimed to assess if mosquitoes colonized with *F*. *tularensis* could transmit these bacteria into sterile nectar after feeding. Mosquitoes were allowed to feed on a nectar surrogate (30% sucrose) inoculated with *F*. *tularensis*, or sterile 30% sucrose as a control. In addition, a sterile 30% sucrose solution (replaced every 24 h) was present and plated on selective media daily to determine if the *F*. *tularensis* could be transmitted by the mosquitoes. Colonies that resembled *F*. *tularensis* color and morphology were recovered from the previously sterile sucrose (in a representative experiment, 23 colonies were recovered on day 2, 18 colonies on day 6) suggesting mosquito transfer. No bacteria were recovered from the sucrose solution fed to mosquitoes in the control group (uninoculated nectar surrogate). After all mosquitoes had perished, we introduced a similar sterile sucrose solution and sampled this for *F*. *tularensis* CFU for three days. No bacteria resembling *F*. *tularensis* were recovered from this material suggesting that the transfer of bacteria to nectar surrogate was dependent on the presence of viable mosquitoes (0 *F*. *tularensis* LVS CFU were recovered over a three-day time period after mosquito mortality).

**Fig 4:**
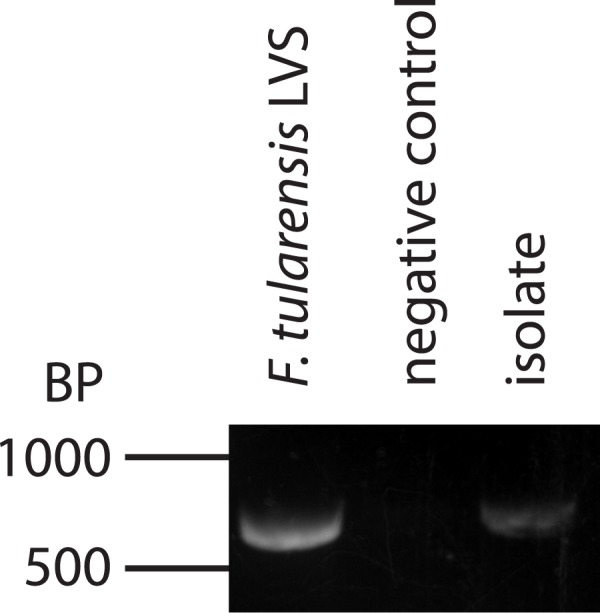
Mosquitoes transfer *F*. *tularensis* from one nectar surrogate source to another. DNA was extracted from bacteria isolated from media selective for *F*. *tularensis*. Only colonies that produced a similar morphology to *F*. *tularensis* were selected. The extracted DNA was subjected to PCR using primers specific for *mglA* of *Francisella* sp. Agarose gel electrophoresis was used to compare amplicons produced from bacteria isolated from nectar surrogate to those generated from bona fide *F*. *tularensis* DNA. PCR from only one isolate is shown for simplicity, but all other colonies with similar morphologies produced a similar amplicon band. PCR reactions lacking template DNA did not produce amplicons.

As aforementioned, mosquitoes that fed on (interacted with) inoculated sucrose were capable of depositing bacteria that produced colonies similar to the morphology of *F*. *tularensis* LVS. To verify that these bacteria were *F*. *tularensis*, DNA was extracted from these isolates, and this genomic material was subjected to PCR using primers specific for *F*. *tularensis mglA*. The amplicon produced was compared to one generated using bona fide *F*. *tularensis* DNA via gel electrophoresis. PCR using DNA from the isolated colonies produced a band with a similar size to that of the authentic *F*. *tularensis* (Fig 4), whereas the reaction lacking a template did not produce an amplicon. These results suggest that *F*. *tularensis* bacteria were deposited into sterile nectar surrogate by mosquitoes that had fed on previously inoculated sucrose solution. Although these data are not quantitative, they do indicate that *F*. *tularensis* can be transported to/from the nectar surrogate by mosquitoes. Therefore, it is plausible that mosquitoes could transfer *F*. *tularensis* from one source of nectar to another in nature.

## Conclusions

Data presented here suggest that *F*. *tularensis* can survive in flower nectar. Additionally, mosquitoes can uptake *F*. *tularensis* from flower nectar and transmit these bacteria to sterile nectar. These data suggest that flower nectar may be capable of serving as a temporary source of *F*. *tularensis*, which in turn alters the disease transmission paradigm of *F*. *tularensis*. Mosquitoes may feed on an infected mammalian host and subsequently feed on flower nectar before moving on to a new host. As a result, *F*. *tularensis* could be transferred to the nectar. Mosquitoes feeding on this nectar source after bacterial transfer may have the potential to become colonized by *F*. *tularensis*. Thus, flower nectar may allow for vector-vector transmission of *F*. *tularensis* without the need of a mammalian host intermediate. In regions containing high mosquito-associated tularemia outbreaks, future work should determine whether flower nectar contains viable *F*. *tularensis*. Data presented in this manuscript represent work modeled in a laboratory using a sister-species to the *Aedes* mosquitoes normally associated with tularemia outbreaks. Moreover, due to lack of availability of authentic nectar, the transmission studies were carried out using a sucrose solution that was not equivalent at maintaining *F*. *tularensis* viability. Finally, lack of quantitative data in this study does not allow for us to extrapolate or make predictions about disease dissemination in nature. Future studies should focus on better recreating plant nectar, and the utilization of mosquito species native to Sweden.

*F*. *tularensis* has been shown to persist in aquatic environments and in species of protozoa [30, 31]. Additionally, mosquito larvae exposed to *F*. *tularensis* in water maintain the bacterium to adulthood [18, 21]. This phenomenon has been shown to be capable of causing mammalian infection [18]. Our results could suggest that it may also be possible for mosquitoes to transmit *F*. *tularensis* from the aquatic reservoir to nectar, further perpetuating the bacterium in nature. Future investigation is necessary to determine whether aquatic *F*. *tularensis* are capable of being transmitted to nectar by mosquitoes.

Mosquitoes likely contribute to seasonal tularemia outbreaks as previously described in the literature [13, 32, 33]. Increased numbers of infections could correlate with peak mosquito and flower activity in specific geographic regions [13, 16]. These outbreaks would therefore be associated with the flowers present in late summer/ early fall which would include *Lappula deflexa* and *Dianthus arenarius* among others. Future work should investigate whether nectar from these and other late summer/ early fall Scandinavian flowers support the viability of *F*. *tularensis*. Increasing global temperatures may allow for higher quantities of both mosquitoes and nectar-bearing flowers to exist seasonally due to more favorable growth conditions [34, 35]. Therefore, mosquito-borne tularemia, which is largely dependent upon weather patterns, may present an increased abundance in years to come. The mosquito-nectar relationship may present a potential target for tracking and perhaps preventing tularemia outbreaks, especially in Scandinavian countries.

There are several additional unexplored factors that may lead to a better understanding of the disease paradigm of mosquito-borne tularemia. It is still unclear whether mosquitoes should be categorized as mechanical vectors or biological vectors. To resolve this, investigation into whether *F*. *tularensis* is spread externally on the proboscis of the mosquito or internally in the salivary glands would be required. If it is determined that mosquitoes harbor the bacterium internally, it will be important to quantify the amount of time that *F*. *tularensis* persists in the mosquito.

Another point of future investigation would be determining the nutrient content of flower nectar to understand how *F*. *tularensis*, a fastidious organism, is able to survive within this plant niche. Variation in nectar content may make certain flower species more capable reservoirs of *F*. *tularensis*. It would also be interesting to investigate if the presence of *F*. *tularensis* within nectar alters mosquitoes’ preference for feeding from the nectar source [36]. Categorization of endemic flower species may put specific countries or regions at a higher risk of mosquito-borne tularemia outbreaks. Another interesting topic would include sampling nectar sources from areas with frequent tularemia outbreaks, such as Sweden, for the presence of *F*. *tularensis*. The experimental approach described in this manuscript may also be applied to other biting arthropods that exhibit nectar feeding habits similar to mosquitoes, including sand flies and horse flies [37]. Moreover, these findings could have implications in the disease paradigms of other mosquito-borne pathogens.

Here, we presented evidence suggesting that colonized mosquitoes can deposit *F*. *tularensis* into sterile nectar-surrogate solution. As early as three days after the feeding on the sterile nectar surrogate, we were able to detect the transfer of *F*. *tularensis* into this sucrose solution. As mosquitoes are known to feed *en masse* on flower nectar, multiple colonized insects could potentially deposit bacteria within the same flower [38]. As the nectar is continuously produced by the flower, this could allow for the accumulation of bacteria through multiple feedings. We speculate that in certain conditions, this number may be high enough to allow for a mosquito to be newly colonized. Because *F*. *tularensis* has an infectious dose of ~1 CFU [39], acquisition of a single bacterium by a mosquito could potentially lead to downstream infection of a mammal.
